# Relationships Between Retinal Amyloid Imaging and Amyloid PET in the A4 Trial

**DOI:** 10.1002/alz.090880

**Published:** 2025-01-09

**Authors:** Robert A. Rissman, Jennifer Ngolab, Michael C. Donohue, Alison Belsha, Jennifer Salazar, Paula Cohen, Sandhya Jaiswal, Veasna Tan, Neelum T. Aggarwal, Jessica Alber, Ken Johnson, Gregory A Jicha, Christopher H. van Dyck, James J. Lah, Stephen Salloway, Michael Rafii, Paul S. S. Aisen, Reisa A Sperling

**Affiliations:** ^1^ Alzheimer's Therapeutic Research Institute, University of Southern California, San Diego, CA USA; ^2^ Rush University Medical Center, Chicago, IL USA; ^3^ George & Anne Ryan Institute for Neuroscience, Kingston, RI USA; ^4^ University of Rhode Island, Kingston, RI USA; ^5^ Neurovision Inc, Sacramento, CA USA; ^6^ University of Kentucky Sanders‐Brown Center on Aging, Lexington, KY USA; ^7^ Alzheimer's Disease Research Unit, Yale School of Medicine, New Haven, CT USA; ^8^ Emory University, Atlanta, GA USA; ^9^ Butler Hospital and Warren Alpert Medical School of Brown University, Providence, RI USA; ^10^ Alpert Medical School, Brown University, Providence, RI USA; ^11^ Center for Alzheimer’s Research and Treatment, Brigham and Women’s Hospital, Massachusetts General Hospital, Harvard Medical School, Boston, MA USA

## Abstract

**Background:**

Alterations to the retina manifest in patients diagnosed with neurodegenerative diseases such as Alzheimer’s disease (AD). Retinal imaging techniques open the possibility for non‐invasive evaluation of AD pathology. Clinically AD diagnosed patients exhibit retinal amyloid deposits. Few studies monitoring preclinical individuals exist, limiting the assessment of the feasibility of retinal imaging as a biomarker for early‐stage AD risk detection.

**Method:**

We compared retinal and cerebral amyloid in clinically normal individuals who screened positive for amyloid through positron emission tomography (PET) from the Anti‐Amyloid Treatment in Asymptomatic Alzheimer Disease (A4) as well as a companion cohort of individuals who were negative on amyloid by amyloid PET in the Longitudinal Evaluation of Amyloid Risk and Neurodegeneration (LEARN) study. We quantified the number of curcumin‐positive fluorescent retinal spots from a small subset of participants from both studies to determine retinal amyloid deposition at baseline.

**Result:**

Participants from the A4 trial exhibited a greater number of retinal spots compared to those from the LEARN study. We report a positive correlation between retinal spots and brain amyloid, as measured by the standardized uptake value ratio (SUVr).

**Conclusion:**

The results of this small pilot study support the use of retinal fundus imaging for detecting amyloid deposition that is correlated with brain amyloid PET SUVr. A larger sample set is under analysis currently to fully ascertain the relationship between amyloid PET and retinal amyloid both cross‐sectionally and longitudinally.

